# *Tectum opticum* development in embryonic quail *(Coturnix japonica)* and the role of HSP70 in the developmental process

**DOI:** 10.2478/jvetres-2026-0030

**Published:** 2026-06-30

**Authors:** Dilem Gülece Ermutlu, Serap İlhan Aksu, Turgay Deprem, Serap Koral Taşçi, Şahin Aslan, Rumeysa Nur Aslan

**Affiliations:** Department of Histology and Embryology, Faculty of Veterinary Medicine, Kafkas University, 36100 Kars, Türkiye

**Keywords:** embryonic development, histology, HSP70, quail, *tectum opticum*

## Abstract

**Introduction:**

The *tectum opticum*, which is a section of the mesencephalon, is located between the cerebellum and the ventral hemispherium of birds. In birds, the *tectum opticum* has a well-developed, thick, layered structure. It is quite large compared to those of other vertebrates and is considered the primary pathway of visual information to the telencephalon. Heat-shock proteins (HSPs) are molecules that help fold newly synthesised polypeptides, assemble multiprotein complexes and transport proteins across cellular membranes.

**Material and Methods:**

In this study, 50 fertilised quail eggs were used to monitor embryonic development. The fixed tissues were blocked in paraffin, and Crossmonn’s triple stain was applied to these sections for histological examination. The avidin-biotin-peroxidase complex technique was applied to determine the immunohistochemical localisation of HSP70.

**Results:**

Histologically, the *tectum opticum* was observed to consist of six main layers. They were formed starting from embryonic day 7 (E7). Ten sublayers of the *stratum griseum et fibrosum superficiale* layer became clear starting from embryonic day 12. Immunohistochemical staining showed HSP70 immunoreactivity in neuron bodies and nerve fibres starting from E7.

**Conclusion:**

The findings suggest that HSP70 contributes to the protection of cells and the continuity of developmental processes. The regulatory effect of HSP70 on cell cycle, apoptosis and differentiation processes during embryonic development is of vital importance for the healthy development of avian embryos. Therefore, it is clear that the mechanisms of HSP70 expression need to be known.

## Introduction

Various bird species have been used for biological research; for embryonic neural development studies Japanese quail are among the preferred birds (14). The bird brain, which consists of the same components as the mammalian brain, is examined in three parts: the prosencephalon (comprised of the telencephalon and diencephalon), the mesencephalon and the rhombencephalon (with its metencephalon and myelencephalon). The optic lobe (*tectum opticum*), a part of the mesencephalon, is located between the ventral hemispheres and the cerebellum. In birds, the *tectum opticum* is a well-developed, thick, layered structure (5). It is responsible for generating visual and auditory responses to environmental stimuli that may include moving prey or predators and is considered the primary pathway for visual information to the telencephalon (9). In birds, it is large compared to those in other vertebrates. Histological studies have been conducted on the *tectum opticum* in some bird species such as chickens (17, 24), falcons (3), pigeons (18) and nightingales (1) during the post-hatching period. In the pre-hatching period, the cellular structure of the *tectum opticum* has been histologically defined in chickens (34) and helmeted guinea fowl (31), and studies on embryonic developmental stages have been conducted in quail (23, 30). However, a literature search did not reveal any developmental studies on the histological structure of the *tectum opticum* in quail during the embryonic period.

Because the *tectum opticum* undergoes rapid cellular proliferation, differentiation and synaptic organisation during embryogenesis, its development requires tightly regulated intracellular processes. These processes are particularly sensitive to disturbances in protein folding and cellular stress. In this context, molecular chaperones such as the heat-shock proteins (HSPs) are of general relevance to embryonic development. These proteins are important protectors of the development of cells and organs and have been reported to be associated with embryogenesis in vertebrate models (8, 15). Heat-shock protein 70 is stated to be expressed throughout implantation, blastulation, gastrulation, neurulation and organogenesis (27). The protein has been reported to play a protective role for the embryo at the blastula stage during the preservation of fertilised poultry eggs (7), and other heat-shock proteins have been found to be involved in protein folding, assembly of multiple protein complexes and transport of proteins to appropriate locations (29). A wide variety of metabolic events also depend on HSPs, including signal transduction, cell growth, cell proliferation, cell differentiation and apoptosis (10, 21).

Expression of HSPs is known to be induced by stressful stimuli. However, studies on their role in embryonic development in poultry in the absence of heat stress have not been found. It is believed that determining the histological structure of the *tectum opticum* during embryonic development and the immunohistochemical localisation of HSP70 will contribute to the literature.

### Material and Methods

Before starting the study, an application was made to and approval was obtained from the Kafkas University Local Ethics Committee for Animal Experiments (KAU-HADYEK/2024-152).

In this study, 50 fertilised quail eggs were used to monitor embryonic development. The eggs were obtained from Prof. Dr. Ali Rıza Aksoy at the Application and Research Farm of the Faculty of Veterinary Medicine, Kafkas University. The eggs were placed in an incubator (DF-103; Cimuka Kuluçka, Ankara, Türkiye) and kept at 37°C and 70% humidity. The day the eggs were first placed in the incubator was considered the embryonic day 0 (E0). From the 7^th^ day (E7) until the hatching (16^th^) day (E16), embryos were taken from five eggs each day, and their viability was assessed in petri dishes before the embryos were fixed in 10% formaldehyde. The fixed tissues were blocked in paraffin, and 5 μm sections were taken from each block and placed onto chromalum gelatin-coated slides. Crossmonn’s triple stain was applied to these sections for histological examination (11).

The avidin-biotin-peroxidase complex technique (6, 20) was applied to determine the immunohistochemical localisation of HSP70. Sections were incubated overnight at +4°C (22) with anti-HSP70 (Cat. No. sc-24; Santa Cruz Biotechnology, Dallas, TX, USA) used at a dilution of 1:100. After incubation, they were washed with PBS, and secondary antibody (biotinylated goat anti-rabbit from Lab Vision, Fremont, CA, USA) was added. The samples were left at room temperature for 30 min, washed in PBS (3×5 min) and treated with streptavidin horseradish peroxidase and left to stand for 30 min. Mayer’s haematoxylin was used for staining the nuclei. The prepared specimens were examined and photographed under a light microscope (Olympus Bx51, Tokyo, Japan).

The thickness of the *tectum opticum* layers was measured using the ImageJ program, v. 1.52a/Java 1.8.0-112 (33). Measurements were performed on five animals for each incubation day between E7 and E16, resulting in a total of 50 animals being measured. For each animal, 100-μm sections stained with Crossmonn’s triple stain were analysed.

The collected data were analysed using the SPSS program, v. 25.0 (IBM, Armonk, NY, USA), being subjected to one-way ANOVA followed by Tukey and Duncan tests for multiple comparisons. The significance levels of the differences between the mean values of the groups were determined, with a P-value < 0.05 considered significant.

## Results

### Histological analysis results

**Fig 1.**
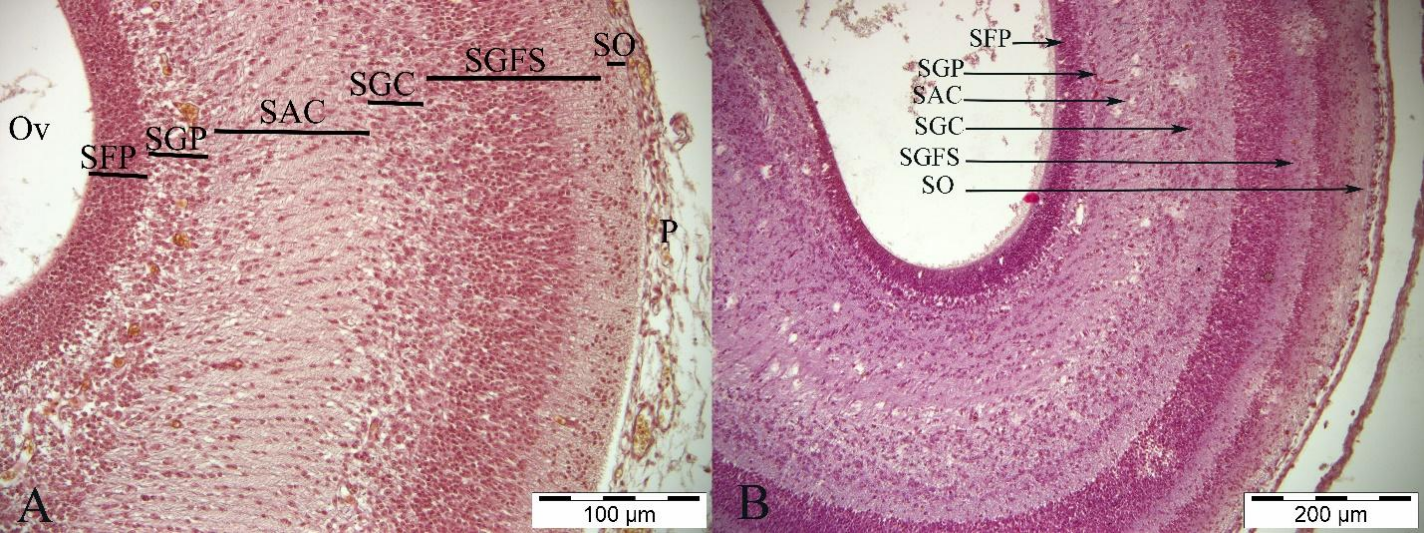
Histological structure of a Japanese quail *tectum opticum* on embryonic days 7 (E7) and 9 (E9). A – E7; B – E9. SO – *stratum opticum*; SGFS – *stratum griseum et fibrosum superficiale*; SGC – *stratum griseum centrale*; SAC – *stratum album centrale*; SGP – *stratum griseum periventriculare*; SFP – *stratum fibrosum periventriculare*; Ov – optic ventricle; P – pia mater. Visualised with Crossmonn’s modified triple staining

Histological examinations revealed that the *tectum opticum* was surrounded by a membrane composed of mesenchymal connective tissue and rich in blood vessels. It was determined that six basic layers formed starting from E7. From outside to inside, these layers were the *stratum opticum* (SO), *stratum griseum et fibrosum superficiale* (SGFS), *stratum griseum centrale* (SGC), *stratum album centrale* (SAC), *stratum griseum periventriculare* (SGP) and *stratum fibrosum periventriculare* (SFP) (Fig. 1A).

### Stratum opticum

**Fig 2.**
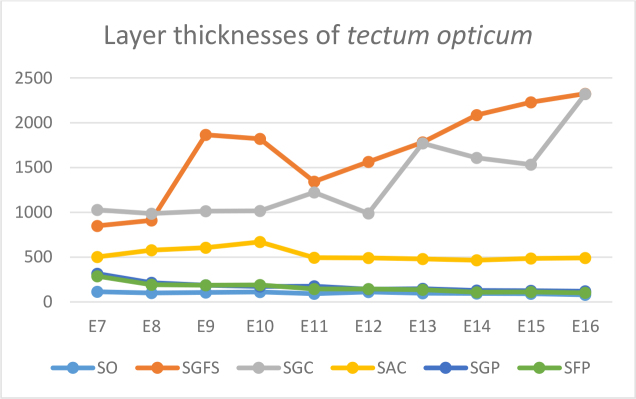
Graphic representation of Japanese quail *tectum opticum* layer thicknesses. E7–16 – embryonic days 7–16; SO – *stratum opticum*; SGFS – *stratum griseum et fibrosum superficiale*; SGC – *stratum griseum centrale*; SAC – *stratum album centrale*; SGP – *stratum griseum periventriculare*; SFP – *stratum fibrosum periventriculare*

It is the layer located beneath the pia mater, consisting of a low number of small spherical neurons, myelinated nerve fibres, retinal fibres and glial cells. It was observed that the SO and SGFS layers were not clearly separated by boundaries during the first days of incubation, but that the boundary between these layers became more distinct from day E9 onwards (Fig. 1B). The thickness of the SO was found to decrease significantly (P-value < 0.05) in the later days of incubation (Table 1, Fig. 2).

**Table 1.** *Tectum opticum* layer thickness measurements in Japanese quail between embryonic days 7 (E7) and 16 (E16)

	SO	SGFS	SGC	SAC	SGP	SFP
E7	113.7±12.7^a^	849.2±41.8^a^	1028.1±112.4^a^	503.5±38.5^a^	316.4±60.9^a^	287.6±37.2^a^
E8	99.2±13.1^b^	910.7±52.5^a^	986.22±103.8^a^	578.63±51.8^b^	215.92±28.1^b^	192.11±41.2^b^
E9	106.6±19.8^a^	1865.4±65.5^b^	1012.74±102.5^a^	606.2±40.5^b^	185.9±19.8^b^	187.2±19.6^b^
E10	111.15±9.3^a^	1820±189.8^b^	1015.5±87.8^a^	669.61±85.2^c^	171.63±19.3^b^	189.44±22.7^b^
E11	92.62±9.1^b^	1343.9±55.9^c^	1222.31±103.3^a^	494.82±50.9^a^	174.88±26.8^c^	143.51±21.1^c^
E12	112.1±9.4^a^	1562.2±39.9^c^	987.3±93.7^a^	490.32±39.2^a^	140.7±16.9^d^	145.3±33.2^d^
E13	97.4±10.2^b^	1780.2±65.6^b^	1770.9±88.6^b^	478.9±68.2^a^	148.3±21.1^c^	137.23±26.7^c^
E14	93.4±14.4^b^	2085.3±105.8^d^	1607.82±186.1^b^	465.4±48.2^a^	128.44±23.5^d^	109.88±11.9^d^
E15	91.7±11.9^b^	2226.8±102.8^d^	1532.8±161.4^b^	486.4±52.3^a^	125.63±15.5^d^	111.17±16.8^d^
E16	79.4±13.2^d^	2324.11±111.5^d^	2318.73±120.1^c^	490.71±3.1^a^	119.75±14.9^d^	101.64±15.4^d^

1 SO – *stratum opticum*; SGFS – *stratum griseum et fibrosum superfciale*; SGC – *stratum griseum centrale*; SAC – *stratum album centrale*; SGP – *stratum griseum periventriculare*; SFP – *stratum fibrosum periventriculare*;

a, b, c, d –different superscript letters in the same row indicate statistically significant difference

### Stratum griseum et fibrosum superficiale

**Fig 3.**
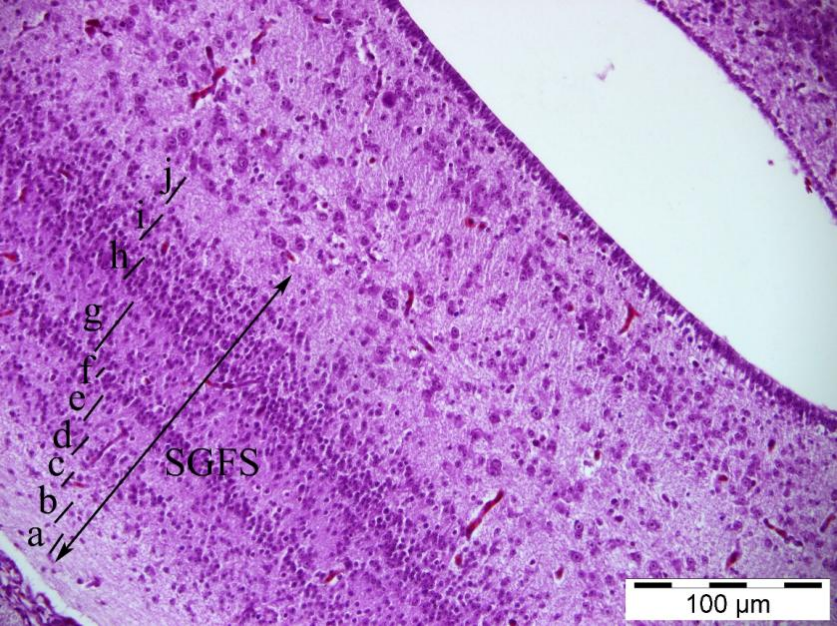
Histological structure of the *stratum griseum et fibrosum superficiale* (SGFS) layer of a Japanese quail *tectum opticum* on embryonic day 12. a–j – sublayers from deep to superficial. Visualised with Crossmonn’s modified triple staining

This next layer is the most prominent one in the *tectum opticum* and consists of small spherical neurons. Ten sublayers of the SGFS (Fig. 3) were possible to distinguish starting from E12, and were designated a, b, c, d, e, f, g, h and j from deep to superficial. Of these sublayers, a, b, c, e, g and j were found to be poor in cells but rich in nerve fibres. Layers d, f, h and i were found to be rich in cells. It was noted that the thickness of the SGFS increased significantly (P-value < 0.05) in the later days of incubation, and that the cell density in this layer decreased towards the end of incubation (Table 1, Fig. 2).

### Stratum griseum centrale

**Fig 4.**
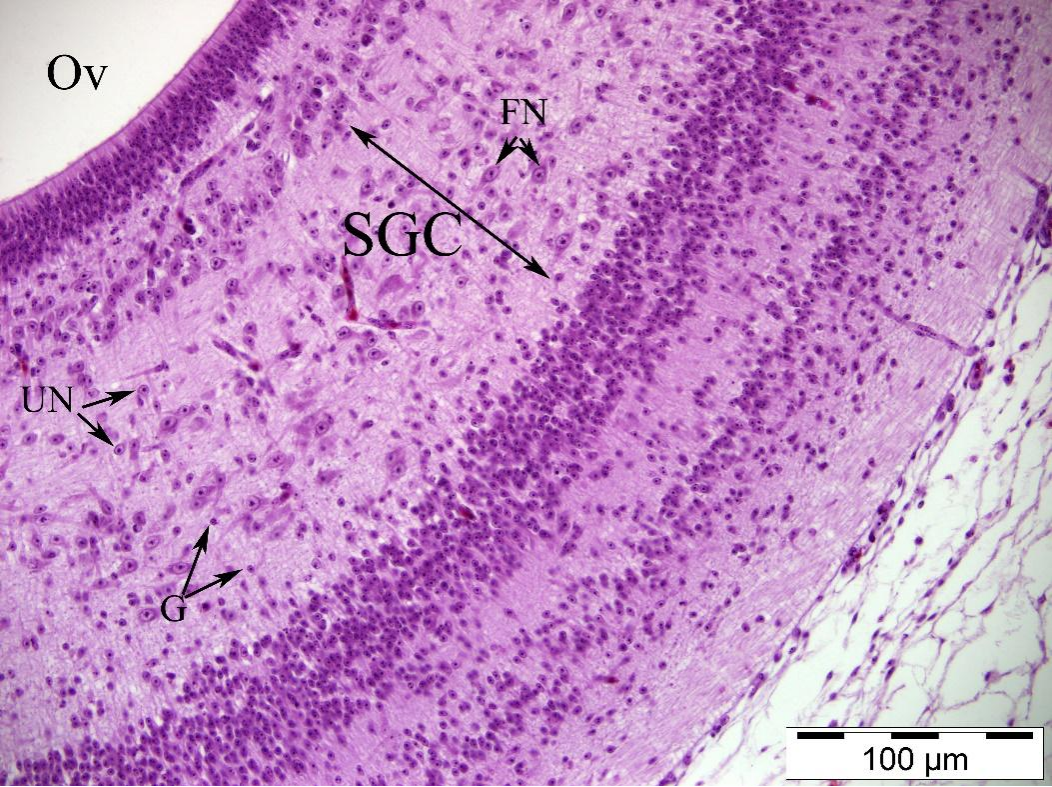
Neurons in the *stratum griseum centrale* (SGC) layer of a Japanese quail *tectum opticum* on embryonic day 11. Ov – optic ventricle; UN – unipolar neuron; G – glial cells; FN – fusiform neuron. Crossmonn’s modified triple staining

This layer, which follows the SGFS, was found to consist of large round neurons. From E10 onwards, in addition to spherical neurons, fusiform, unipolar and pyramidal neurons were also observed in this layer (Fig. 4). The thickness of the SGC was found to increase significantly (P-value < 0.05) in the later days of incubation (Table 1, Fig. 2).

### Stratum album centrale

Spherical shaped neurons and glial cells were observed in the SAC (Fig. 1). The thickness of the stratum album centrale was found to decreased significantly (P-value < 0.05) in the later days of incubation (Table 1, Fig. 2).

### Stratum griseum periventriculare

**Fig 5.**
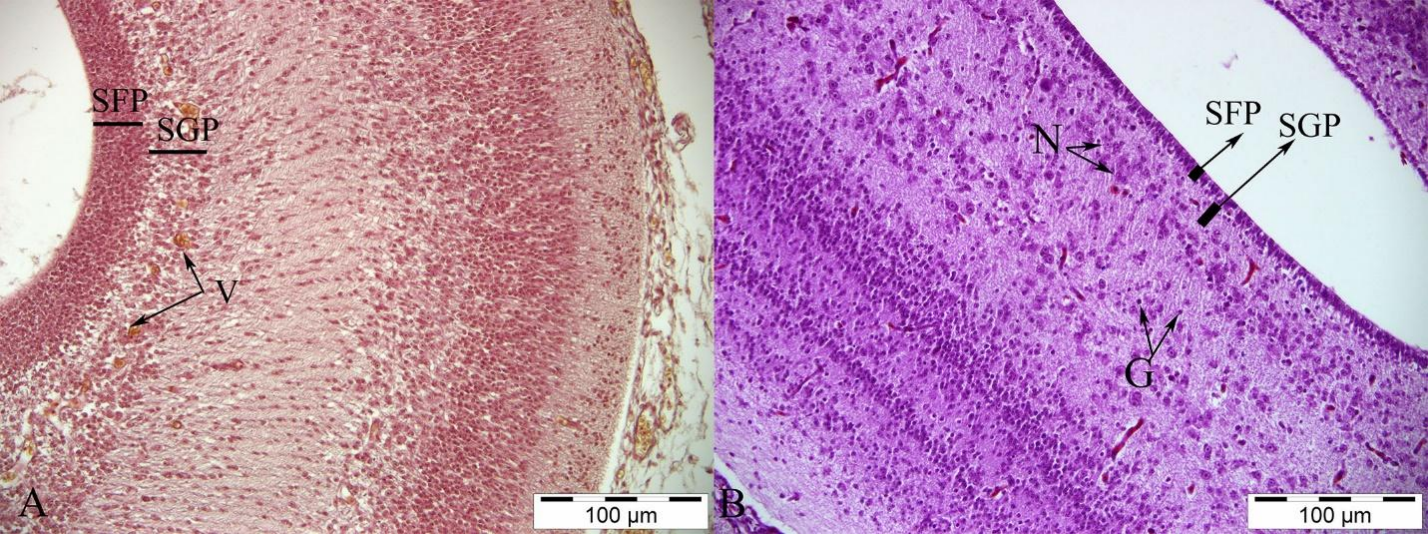
Histological structure of the *stratum griseum periventriculare* (SGP) and *stratum fibrosum periventriculare* (SFP) layers of a Japanese quail *tectum opticum* on embryonic days 7 (E7) and 12 (E12). A – E7; B – E12. N – spherical neuron; V – blood vessel; G – glial cells. Visualised with Crossmonn’s modified triple staining

Sphericalshaped neurons and glial cells were observed in the SGP. Abundant blood vessels were seen in this layer on E7 (Fig. 5). The thickness of the SGP was found to decrease significantly (P-value < 0.05) in the later days of incubation (Table 1, Fig. 2).

### Stratum fibrosum periventriculare

This fibrous layer surrounds the optic ventricle, along with ependymal cells that have cuboidal epithelia (Fig. 5). It was observed that the ependymal cells and the SFP layer were not separated by distinct boundaries. The thickness of this layer, which is densely populated with neuroepithelial cells, was significantly decreased (P-value < 0.05) between E7 and E16 (Table 1, Fig. 2).

### Immunohistochemical results

**Fig 6.**
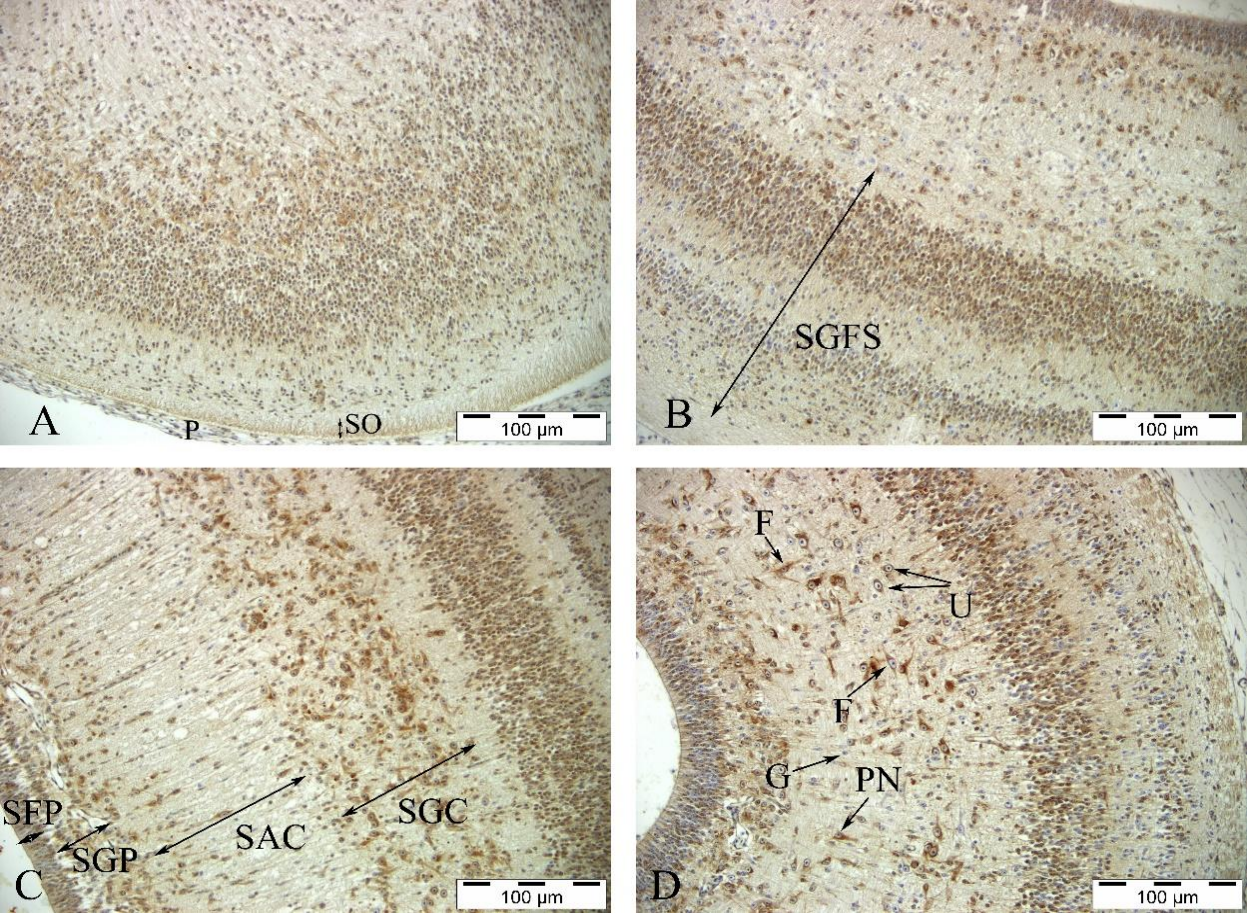
Heat-shock protein 70 immunostaining in a Japanese quail *tectum opticum*. A – on embryonic (E) day 7; B – E9; C – E10; D – E11. SO – *stratum opticum*; SGFS – *stratum griseum et fibrosum superfiiciale*; SFP – *stratum fibrosum perivenriculare*; SGP – *stratum griseum periventriculare*; SAC – *stratum album centrale*; SGC – *stratum griseum centrale*; P – pia mater; F – fusiform neuron; G – glial cells; PN – pyramidal neuron; U – unipolar neuron

**Fig 7.**
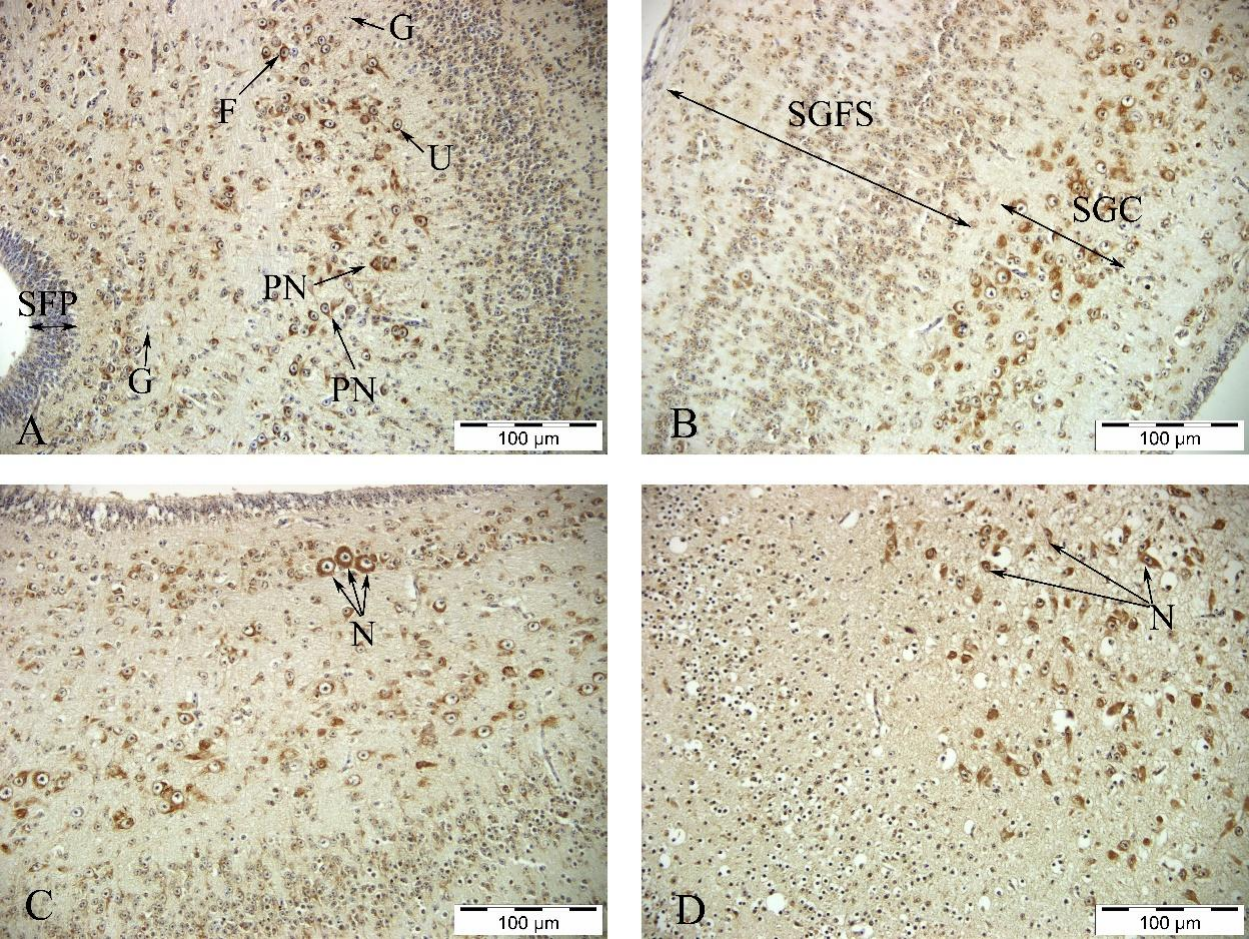
Heat-shock protein 70 immunostaining in a Japanese quail *tectum opticum*. A – on embryonic (E) day 13; B – E14; C – E15; D – E16. SFP – *stratum fibrosum perivenriculare*; SGFS – *stratum griseum et fibrosum superficiale*; SGC – *stratum griseum centrale*; G – glial cells; F – fusiform neuron; PN – pyramidal neuron; U – unipolar neuron; N – neurons showing high immunoreactivity

Immunohistochemical examinations revealed that HSP70 exhibited immunoreactivity in neuron cell bodies, extensions and nerve fibres. No reaction was observed in neuroglial cells (Figs 6 and 7).

### Stratum opticum

In this layer consisting of nerve fibres, weaker HSP70 immunoreactivity was observed in E7 and E11 compared to its immunoreactivity in the other layers (Fig. 6).

### Stratum griseum et fibrosum superficiale

Weak HSP70 immunoreactivity was observed in small spherical neurons located in the lower layers where cell density was higher. Immunoreactivity in the SGFS layer, which increased in thickness in parallel with the growth of the *tectum opticum*, decreased from E13 onwards (Figs 6 and 7).

### Stratum griseum centrale

The protein’s immunoreactivity in this layer, where fusiform, spherical, unipolar and pyramidal neurons were located, showed a significant increase from E7 to E16 (Figs 6 and 7).

### Stratum griseum periventriculare

Similar to the immunoreactivity change in the SGC layer, an increase in HSP70 immunoreactivity was detected in neurons between E7 and E16 (Fig. 6).

### Stratum fibrosum perivenriculare

In this layer, where cell density and thickness decreased towards the later days of incubation, a decrease in HSP70 immunoreactivity was observed between E7 and E16 (Figs 6 and 7).

## Discussion

In birds, the *tectum opticum* is a highly developed, layered mesencephalic structure responsible for receiving 90% of the information from the retina and directing visual and auditory head movements (5, 35). Proliferative neuroepithelial cells proliferate in the ventricular layer, after which postmitotic cells migrate to different layers, contributing to the formation of the laminar structure of the *tectum opticum*. Histological studies have been conducted on quail (19), nightingales (1), falcons (3) and chickens (17, 28), and have determined that the tectal structure consists of six main layers. Our study also determined that the quail *tectum opticum* consists of six main layers and does not differ in this respect from that of other avian species. We found these layers to be clearly distinguishable from the seventh day of incubation onwards.

The SGFS layer of the *tectum opticum* is divided into sublayers. The literature reports that this layer consists of nine sublayers in broiler chicks (17), and sixteen layers in nightingales (1), chicken embryos (25), pigeons (18) and quail (19). In our study, it was determined that the SGFS layer consisted of ten sublayers, and these sublayers became clearly defined from the 12^th^ day of incubation onwards.

A notable difference between our results and previous observations concerns neuronal development in layers internal to the SGFS over the incubation period. A study on guinea fowl embryos during incubation reported the presence of fusiform and pyramidal neurons in the SGP, SGC and SAC layers on day 18 of incubation (31). In our study, however, fusiform and pyramidal neurons began to appear in the SGC layer from day 11 of incubation. This difference is thought to be due to the different incubation periods of guinea fowl and quail.

Another disparity between our findings and those made in other poultry is in the nonuniformity of the manner of layer development over the early-life period. It has been reported that the thickness of all layers of the broiler chicken *tectum opticum* increases significantly with age after hatching (17). In our study, while the thickness of the SGFS and SGC layers increased with developmental progression, the thickness of the SO, SAC, SGP and SFP layers decreased. The observed changes in layer thickness suggest that embryonic developmental processes differentially affect individual tectal layers.

Changes in layer thickness are not the only features shaped during embryonic development of the *tectum opticum*. This period also determines the cellular composition and functional organisation of the tissue through tightly regulated processes of proliferation, differentiation and protein homeostasis. Disruptions caused by environmental or physiological stressors may therefore impair the proper establishment of neuronal populations and laminar architecture. In this context, heat-shock proteins, particularly HSP70, play a critical protective role. Luft and Dix (27) reported that HSP70 is essential for protecting developing cells against stressinduced damage during embryogenesis. When cells are exposed to various stress factors, they increase the production of heat-shock proteins. In addition to being induced by pathological conditions such as ischaemia and heat stress, these proteins are also expressed under physiological conditions. In these conditions, they are molecular chaperones that mediate the folding of intracellular proteins. They play a critical role in preventing inappropriate protein assembly, mediating intracellular transport, maintaining proteins in inactive forms and degrading misfolded proteins (32). It has been noted that some HSPs are consistently expressed in the adult nervous system. Heat-shock cognate 70 constitutes 2–3% of the total protein content in the mouse spinal cord (2). Studies exist that show that some HSPs are necessary during certain periods of normal embryonic development. In these studies, HSP70 was reported to be necessary during the normal process of lens development in zebrafish embryos under stress-free conditions (15) and to be effective in the differentiation of postmitotic lens fibres. This protein has also been detected in embryonic chicken (13) and human (4) lens development. In a study on mouse embryos (26), HSP70 was detected in neurons in different regions of the developing brain. Luft and Dix (27) additionally reported that the balance between the cell cycle and apoptosis is essential for maintaining cell number in embryogenesis. The observed presence of HSP70 in the densely neuronal layers of the quail *tectum opticum* suggests roles in neuronal development, prevention of misfolding and transport of proteins to relevant sites. The presence of HSP70 in neuroepithelial cells, particularly during the early stages of embryonic development, suggests a protective effect on mitotic activity.

In chicken embryos, it has been reported that more HSP70 is synthesised in the early embryonic period compared to the later stages (16). In quail embryos, although immunoreactivity was observed to decrease in the SO, SGFS, SAC and SFP layers in the early embryonic period, the increase in immunoreactivity in neurons located in the SGC and SGP layers suggests that HSP70 still played important roles in protecting cells. It is thought that in the first days of embryonic development, when there is intense protein synthesis and neuronal differentiation, HSP70 is effective in axonal transport and neuronal development, preventing the aggregation and denaturation of synthesised proteins, and that in the later days of development, immunoreactivity in the SO, SGFS, SAC and SFP layers weakens in parallel with the slowing of protein synthesis. D’Souza and Brown (12) reported that since neurons are non-mitotic and cannot regenerate, they are vulnerable to damage in the brain, and that high HSP levels may be protective for neurons. The persistence of HSP70 in neurons until the end of the incubation period in this study suggests that its effects carry through the phase of most intensive neurogenesis.

## Conclusion

It was determined that the *tectum opticum* in the quail consists of six main layers from E7 onwards. The SGFS layer showed clearly distinguishable sublayers from the 12^th^ day of incubation. As development progressed, it was determined that the thicknesses of the SGFS and SGC layers increased, while the thicknesses of the SO, SAC, SGP and SFP layers decreased. Heat-shock protein 70, which plays a role in cell development, proliferation and differentiation during the embryonic period of intensive active cell division and protein synthesis, was found to exhibit immunoreactivity in neurons and nerve fibres in the SO, SGFS, SGC, SGP and SFP layers of the quail *tectum opticum*. This result indicates that HSP70 is a key part of responses to developmental stresses. The determination of HSP70’s immunoreactivity, particularly in neurons, suggests that it suppresses apoptosis, removes misfolded proteins and maintains intracellular stability. The data obtained in this study reveal that HSP70 functions as a molecular regulator supporting neuronal differentiation and cell survival, rather than as a general stress response in embryonic *tectum opticum* development.
